# Bioinformatics analysis of differentially expressed genes in rotator cuff tear patients using microarray data

**DOI:** 10.1186/s13018-018-0989-5

**Published:** 2018-11-13

**Authors:** Yi-Ming Ren, Yuan-Hui Duan, Yun-Bo Sun, Tao Yang, Meng-Qiang Tian

**Affiliations:** Department of Joint and Sport Medicine, Tianjin Union Medical Center, Jieyuan Road 190, Hongqiao District, Tianjin, 300121 People’s Republic of China

**Keywords:** Rotator cuff muscle, Satellite cells, Differentially expressed genes, Bioinformatics analysis, Calcium signaling, Denervation

## Abstract

**Background:**

Rotator cuff tear (RCT) is a common shoulder disorder in the elderly. Muscle atrophy, denervation and fatty infiltration exert secondary injuries on torn rotator cuff muscles. It has been reported that satellite cells (SCs) play roles in pathogenic process and regenerative capacity of human RCT via regulating of target genes. This study aims to complement the differentially expressed genes (DEGs) of SCs that regulated between the torn supraspinatus (SSP) samples and intact subscapularis (SSC) samples, identify their functions and molecular pathways.

**Methods:**

The gene expression profile GSE93661 was downloaded and bioinformatics analysis was made.

**Results:**

Five hundred fifty one DEGs totally were identified. Among them, 272 DEGs were overexpressed, and the remaining 279 DEGs were underexpressed. Gene ontology (GO) and pathway enrichment analysis of target genes were performed. We furthermore identified some relevant core genes using gene–gene interaction network analysis such as GNG13, GCG, NOTCH1, BCL2, NMUR2, PMCH, FFAR1, AVPR2, GNA14, and KALRN, that may contribute to the understanding of the molecular mechanisms of secondary injuries in RCT. We also discovered that GNG13/calcium signaling pathway is highly correlated with the denervation atrophy pathological process of RCT.

**Conclusion:**

These genes and pathways provide a new perspective for revealing the underlying pathological mechanisms and therapy strategy of RCT.

## Introduction

The rotator cuff muscle complex of the shoulder is comprised of four distinct muscles (supraspinatus, infraspinatus, teres minor, and subscapularis), which controls essential shoulder movements [1, 2]**.** The rotator cuff tear (RCT) is a common cause of impact pain, nocturnal pain and shoulder joint dysfunction, which seriously affect the life and working ability of patients, and reduce the quality of life of patients [3, 4]**.** Most tears require reparative surgery; however, recurrence of tears following surgery is common, with failure rates ranging from 30 to 94% [5]**.** Rotator cuff tendon tears are accompanied by secondary changes in the rotator cuff muscles, including muscle atrophy, denervation, and fatty infiltration, which may explain the progressive loss of function after an acute injury and also the high rate of surgical failure. However, the underlying mechanism is not well understood.

Satellite cells (SCs) are mitotically quiescent muscle stem cells located between the basal lamina and the muscle membrane, which are known to play a key role in the adaptive response of muscle to exercise, and in the maintenance of the regenerative capacity of muscle. Hepatocyte growth factor (HGF) and nitric oxide (NO) could regulate transit of a SC from the quiescent G0 state into the G1 (activated) stage of the cell cycle [6]**.** Recently, Deanna et al. discovered possible supraspinatus denervation in RCT and suggested NO-donor treatment combined with stretching has potential to promote growth in atrophic supraspinatus muscle after RCT and improve functional outcome [7, 8]**.** Lundgreen et al. showed patients with full-thickness tears had a reduced density of SCs, fewer proliferating cells, and atrophy of myofibers [9]**.** With muscle atrophy, fatty infiltration into skeletal muscles is thought to cause muscle degeneration by impairing the myogenic function of SCs [10]**.**

Here, we downloaded the gene expression profile GSE93661 from the Gene Expression Omnibus database (GEO) and made bioinformatics analysis to investigate differentially expressed genes (DEGs) of SCs that regulated between torn supraspinatus (SSP) samples and intact subscapularis (SSC) samples from RCT patients. By doing this, we hope that the key target genes and pathways involved in the pathological process of RCT could be identified and existing molecular mechanisms could be revealed.

## Materials and methods

### Gene expression microarray data

The gene expression profile GSE93661 was downloaded from the Gene Expression Omnibus (GEO, www.ncbi.nlm.nih.gov/geo/). GSE93661 was based on Agilent-026652 Whole Human Genome Microarray 4x44K v2 platform. GSE93661 dataset contained four samples, including two torn SSP samples, and two intact SSC samples.

### DEGs in torn SSP and intact SSC samples

The raw data files used for the analysis included TXT files. The analysis was carried out using GEO2R, which can perform comparisons on original submitter-supplied processed data tables using the GEO query and limma R packages from Bioconductor project. The *P* value < 0.05 and log fold change (FC) > 2.0 or log FC < − 2.0 were used as the cut-off criteria. The DEGs with statistical significance between the torn SSP samples and intact SSC samples were selected and identified.

### GO and KEGG analysis of DEGs

Target genes list were submitted to the DAVID 6.8 (https://david.ncifcrf.gov/tools.jsp) and ClueGO version 2.33 (based on Cytoscape software version 3.4.0 (www.cytoscape.org)) to identify overrepresented GO categories and pathway categories. Gene ontology (GO) analysis was used to predict the potential functions of the DEGs in biological process (BP), molecular function (MF), and cellular component (CC). The Kyoto Encyclopedia of Genes and Genomes (KEGG) is a knowledge base for systematic analysis of gene functions, linking genomic information with higher-level systemic functions. Finally, the overrepresented pathway categories were considered statistically significant using KEGG pathway enrichment analysis.

### Gene interaction network construction

A large number of DEGs we obtained may be RCT-associated genes, and it is suggested that these DEGs in torn SSP samples may participate in the progression of RCT. Firstly, DEGs list was submitted to the Search Tool for the Retrieval of Interacting Genes (STRING) database (http://www.string-db.org/), and an interaction network chart with a combined score > 0.4 was saved and exported. Subsequently, the interaction regulatory network of RCT-associated genes was visualized using Cytoscape software version 3.4.0. The distribution of core genes in the interaction network was made by NetworkAnalyzer in Cytoscape. Then, the plugin Molecular Complex Detection (MCODE) was applied to screen the modules of the gene interaction network in Cytoscape. Venn diagram was drawn using Venny 2.1 (http://bioinfogp.cnb.csic.es/tools/venny/).

## Result

### Identification of DEGs

The gene expression profile GSE93661 was downloaded from the GEO, and the GEO2R method was used to identify DEGs in torn SSP samples compared with intact SSC samples. *P* value < 0.05, log FC > 2.0, or log FC < − 2.0 were used as the cut-off criteria. After analyzing, differentially expression gene profiles were obtained. Totally, 551 DEGs were identified including 272 upregulated DEGs and 279 downregulated DEGs screened in torn SSP samples compared with intact SSC samples. Parts of DEGs were listed in Table 1.

**Table 1:** The top 10 Regulated DEGs in SCs of torn rotator cuff muscle with *P* value< 0.05

ID	*P* value	logFC	Gene symbol
Upregulated			
9522	0.0024644	5.71	FAM196B
39,990	0.0031637	5.05	SCN2A
8187	0.0000663	4.91	NNT
41,585	0.0035824	4.74	TDRD3
8204	0.0040603	4.46	LOC100131129
28,406	0.0002489	4.42	C8orf42
8118	0.0041306	4.42	KIAA1751
12,220	0.0043186	4.32	LRRC2
7774	0.0044309	4.27	CYP2E1
37,687	0.0007211	4.24	CUBN
Downregulated			
32,273	0.0024714	− 4.62	PTPRC
45,097	0.0037977	− 4.61	TCF7L2
12,505	0.0016694	− 4.49	tcag7.1023
35,725	0.0001639	− 4.11	FAM101A
45,093	0.0083048	− 4.08	C8orf67
8427	0.0005044	− 3.84	PTCH2
38,947	0.0004951	− 3.6	PLLP
31,098	0.0001216	− 3.55	TRPV5
31,980	0.0064577	− 3.54	SPNS3
29,856	0.0004283	− 3.52	ACP2

*SCs* satellite cells, *DEGs* differentially expressed genes, *FC* fold change

### GO term enrichment analysis of DEGs

**Fig. 1:**
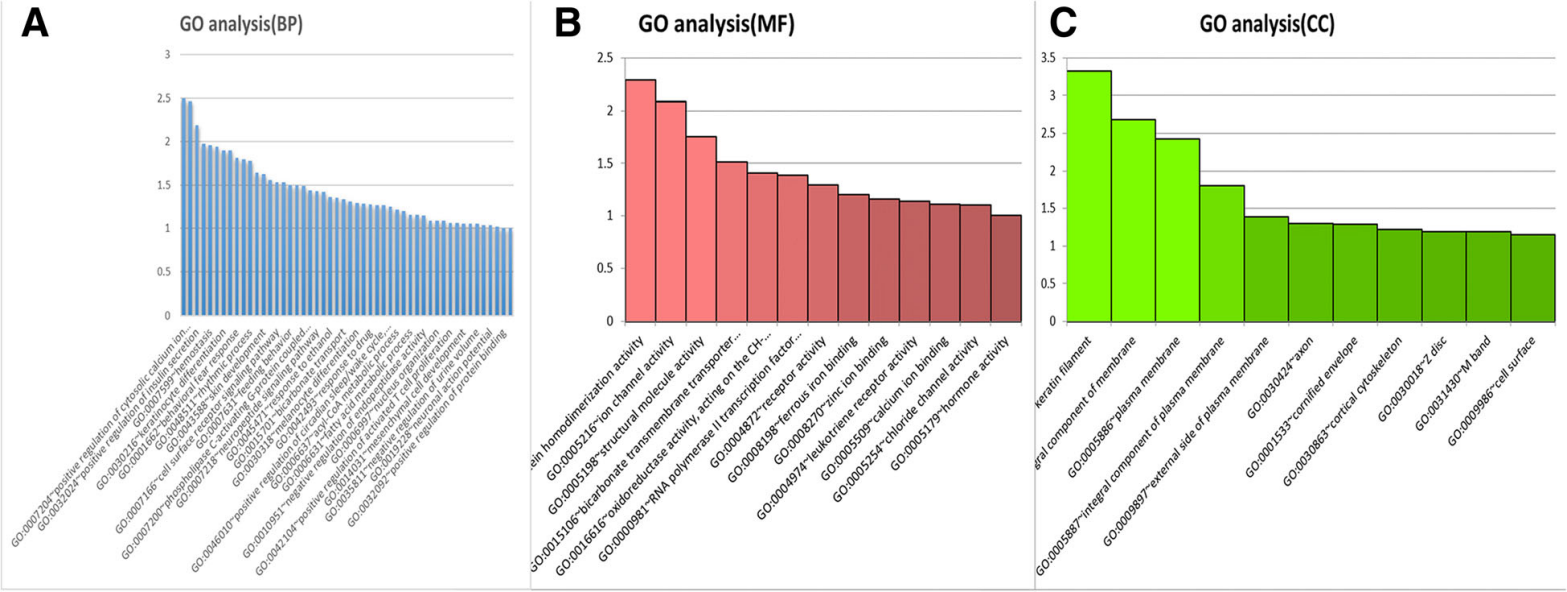
Gene ontology (GO)-enrichment analysis of biological processes (**a**) molecular functions (**b**) and cellular components (**c**). The labels in *Y* axis mean enrichment score (−log_10_ *P* value), and labels in *X* axis mean GO terms

Functional annotation of the 551 DEGs was clarified using the DAVID 6.8 online tool. GO analysis indicated that these DEGs were significantly enriched in muscle contraction, aging, regulation of ion transmembrane transport, mesenchymal cell development, and other biological processes (Fig. 1). For MF, the DEGs were enriched in ion channel activity, calcium ion binding, structural molecule activity, and others. In addition, GO CC analysis also showed that the DEGs were significantly enriched in keratin filament, integral component of plasma membrane, axon, cornified envelope, cortical cytoskeleton, and others.

### KEGG pathway analysis of DEGs

**Fig. 2:**
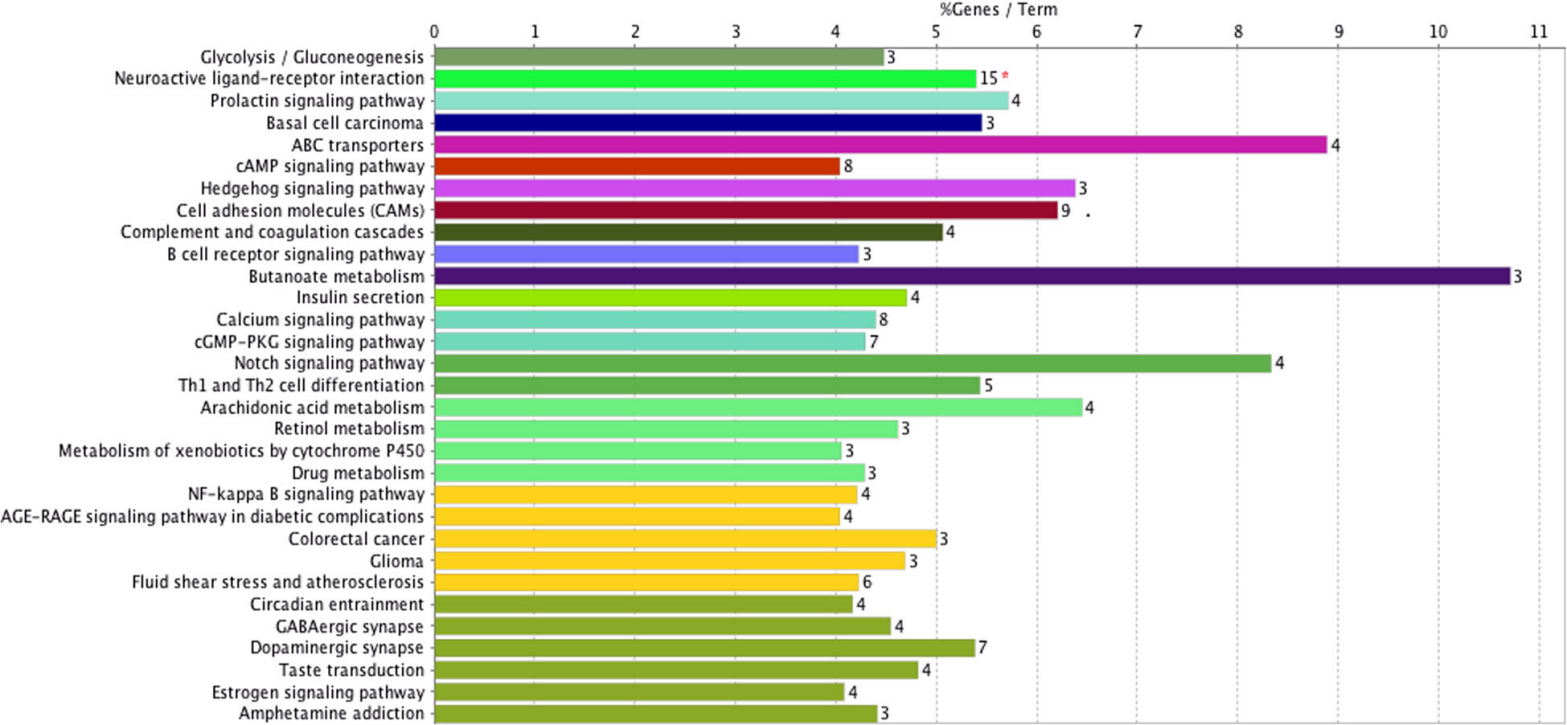
Kyoto Encyclopedia of Genes and Genomes (KEGG) pathway analysis of differentially expressed genes (DEGs). The different colors mean different pathways, and the closer the colors are, the closer the function clustering of pathways are

The result of KEGG pathway analysis revealed that target genes were enriched in butanoate metabolism, ABC transporters, notch signaling pathway, arachidonic acid metabolism, hedgehog signaling pathway, cell adhesion molecules (CAMs), prolactin signaling pathway, neuroactive ligand-receptor interaction, dopaminergic synapse, GABAergic synapse, calcium signaling pathway, cGMP-PKG signaling pathway, drug metabolism, B cell receptor signaling pathway, NF-kappa B signaling pathway, estrogen signaling pathway, cAMP signaling pathway, and others. These key pathways were showed in Fig. 2. Besides, these core pathways and their associated genes found were summarized in Table 2. The first-ranking butanoate metabolism signaling pathway had the 10.71% associated genes, which included ACSM2B, ACSM4, and ACSM6. The second-placed ABC transporters signaling pathway had the 8.89% associated genes, which included ABCA3, ABCD1, ABCG2, and ABCG4. The third-placed notch signaling pathway had the 8.33% associated genes, which included JAG2, MAML3, MFNG, and NOTCH1.

**Table 2:** Core pathways and their associated genes found

GOID	GO term	Term *P* value	% associated genes	Associated genes found
GO:0000650	Butanoate metabolism	0.02	10.71	[ACSM2B, ACSM4, ACSM6]
GO:0002010	ABC transporters	0.01	8.89	[ABCA3, ABCD1, ABCG2, ABCG4]
GO:0004330	Notch signaling pathway	0.02	8.33	[JAG2, MAML3, MFNG, NOTCH1]
GO:0000590	Arachidonic acid metabolism	0.04	6.45	[CYP2B6, CYP2E1, PTGS1, TBXAS1]
GO:0004340	Hedgehog signaling pathway	0.07	6.38	[BCL2, IHH, PTCH2]
GO:0004514	Cell adhesion molecules (CAMs)	0.00	6.21	[CD22, CD226, CD86, CLDN15, ITGAM, NCAM2, NLGN1, PTPRC, VCAM1]
GO:0004917	Prolactin signaling pathway	0.05	5.71	[CISH, MAPK10, PRL, TH]
GO:0005217	Basal cell carcinoma	0.10	5.45	[PTCH2, TCF7L2, WNT10A]
GO:0004658	Th1 and Th2 cell differentiation	0.04	5.43	[IL12RB1, JAG2, MAML3, MAPK10, NOTCH1]
GO:0004080	Neuroactive ligand-receptor interaction	0.00	5.40	[AVPR2, CHRNB3, CHRNE, CYSLTR1, EDNRB, GABBR1, GIPR, GLP2R, HRH3, LTB4R, NMUR2, NPY2R, NPY4R, OPRL1, PRL]
GO:0004728	Dopaminergic synapse	0.02	5.38	[CACNA1C, CALML6, GNG13, KCNJ6, MAPK10, SCN1A, TH]
GO:0004610	Complement and coagulation cascades	0.07	5.06	[F7, ITGAM, MASP2, VWF]
GO:0005210	Colorectal cancer	0.12	5.00	[BCL2, MAPK10, TCF7L2]
GO:0004742	Taste transduction	0.09	4.82	[CACNA1C, GABBR1, GNG13, SCN2A]
GO:0004911	Insulin secretion	0.09	4.71	[CACNA1C, FFAR1, GCG, KCNU1]
GO:0005214	Glioma	0.14	4.69	[CALML6, PDGFB, PLCG2]
GO:0000830	Retinol metabolism	0.14	4.62	[ADH6, CYP2B6, DHRS4L1]
GO:0004727	GABAergic synapse	0.10	4.55	[CACNA1C, GABBR1, GNG13, KCNJ6]
GO:0000010	Glycolysis/gluconeogenesis	0.15	4.48	[ACSS1, ADH6, LDHC]
GO:0005031	Amphetamine addiction	0.16	4.41	[CACNA1C, CALML6, TH]
GO:0004020	Calcium signaling pathway	0.03	4.40	[ATP2A1, ATP2B3, CACNA1C, CALML6, CYSLTR1, EDNRB, GNA14, PLCG2]
GO:0004022	cGMP-PKG signaling pathway	0.05	4.29	[ATP2A1, ATP2B3, CACNA1C, CALML6, EDNRB, KCNU1, NPPB]
GO:0000982	Drug metabolism	0.17	4.29	[ADH6, CYP2B6, CYP2E1]
GO:0004662	B cell receptor signaling pathway	0.17	4.23	[CD22, PLCG2, RASGRP3]
GO:0005418	Fluid shear stress and atherosclerosis	0.07	4.23	[BCL2, CALML6, MAPK10, PDGFB, PIAS4, VCAM1]
GO:0004064	NF-kappa B signaling pathway	0.12	4.21	[BCL2, PIAS4, PLCG2, VCAM1]
GO:0004713	Circadian entrainment	0.13	4.17	[CACNA1C, CALML6, GNG13, KCNJ6]
GO:0004915	Estrogen signaling pathway	0.14	4.08	[CALML6, GABBR1, HSPA6, KCNJ6]
GO:0000980	Metabolism of xenobiotics by cytochrome P450	0.19	4.05	[ADH6, CYP2B6, CYP2E1]
GO:0004024	cAMP signaling pathway	0.06	4.04	[ATP2B3, CACNA1C, CALML6, FXYD1, GABBR1, GHRL, GIPR, MAPK10]
GO:0004933	AGE-RAGE signaling pathway in diabetic complications	0.14	4.04	[BCL2, MAPK10, PLCG2, VCAM1]

### Interaction network of DEGs and core genes in the interaction network

**Fig. 3:**
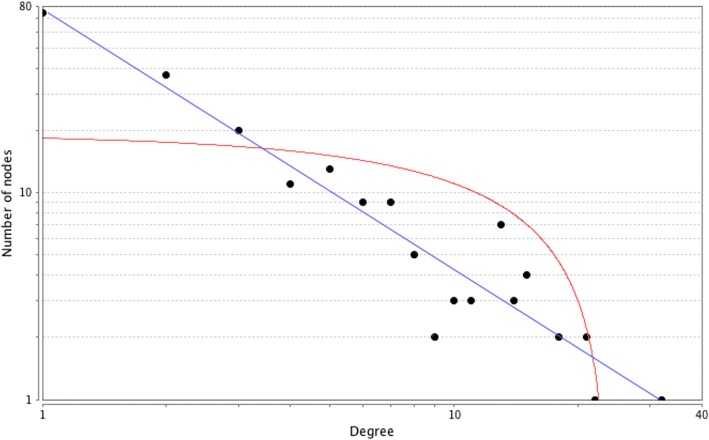
The distribution of core genes in the interaction network. The black node means the core gene. The red line mans the fitted line, and the blue line means the power law. The correlation between the data points and corresponding points on the line is approximately 0.993. The *R*^2^ value is 0.902, giving a relatively high confidence that the underlying model is indeed linear

**Fig. 4:**
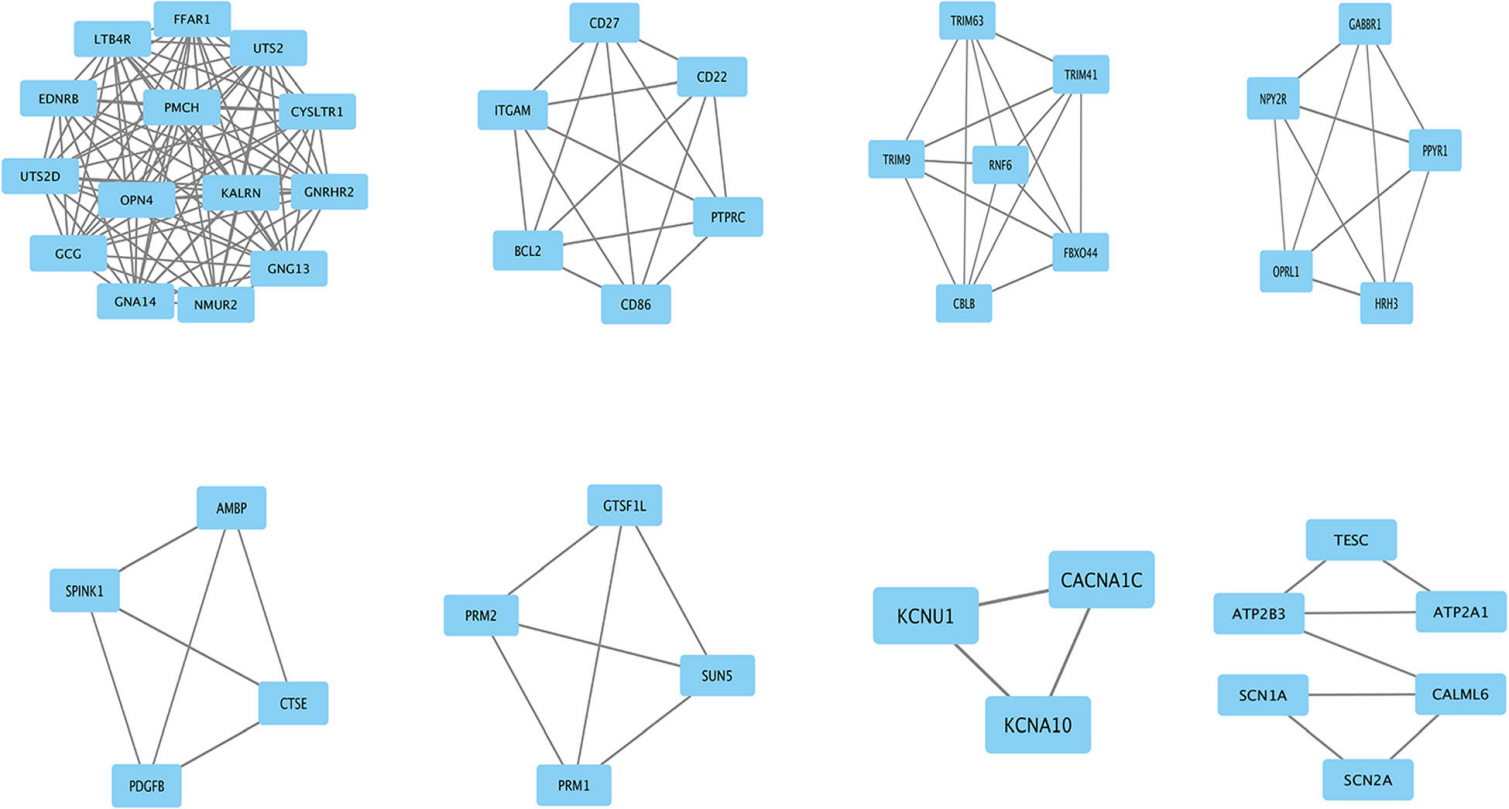
The top 8 modules from the gene–gene interaction network. The squares represent the differentially expressed genes (DEGs) in modules, and the lines show the interaction between the DEGs

Based on the information in the STRING database, the gene interaction network contained 386 nodes and 440 edges. The nodes indicated the DEGs, and the edges indicated the interactions between the DEGs. NetworkAnalyzer in Cytoscape software was used to analyze these genes, and core genes were ranked according to the predicted scores. The top 10 high-degree hub nodes included GNG13, GCG, NOTCH1, BCL2, NMUR2, PMCH, FFAR1, AVPR2, GNA14, and KALRN. Among these genes, GNG13 showed the highest node degree which was 32. The core genes and their corresponding degree were shown in Table 3. The distribution of core genes in the interaction network was revealed in Fig. 3. The correlation between the data points and corresponding points on the line is approximately 0.993. The *R*^2^ value is 0.902, giving a relatively high confidence that the underlying model is indeed linear. Then, we used MCODE to screen the modules of the gene interaction network, and eight modules were showed in Fig. 4.

**Table 3:** The core genes and their corresponding degree

Gene	Degree	Gene	Degree	Gene	Degree	Gene	Degree
GNG13	32	PMCH	18	PTPRC	14	MPO	13
GCG	22	FFAR1	15	CYSLTR1	14	OPN4	13
NOTCH1	21	AVPR2	15	EDNRB	14	GNRHR2	13
BCL2	21	GNA14	15	UTS2D	13	LTB4R	13
NMUR2	18	KALRN	15	UTS2	13	PRL	13

**Fig. 5:**
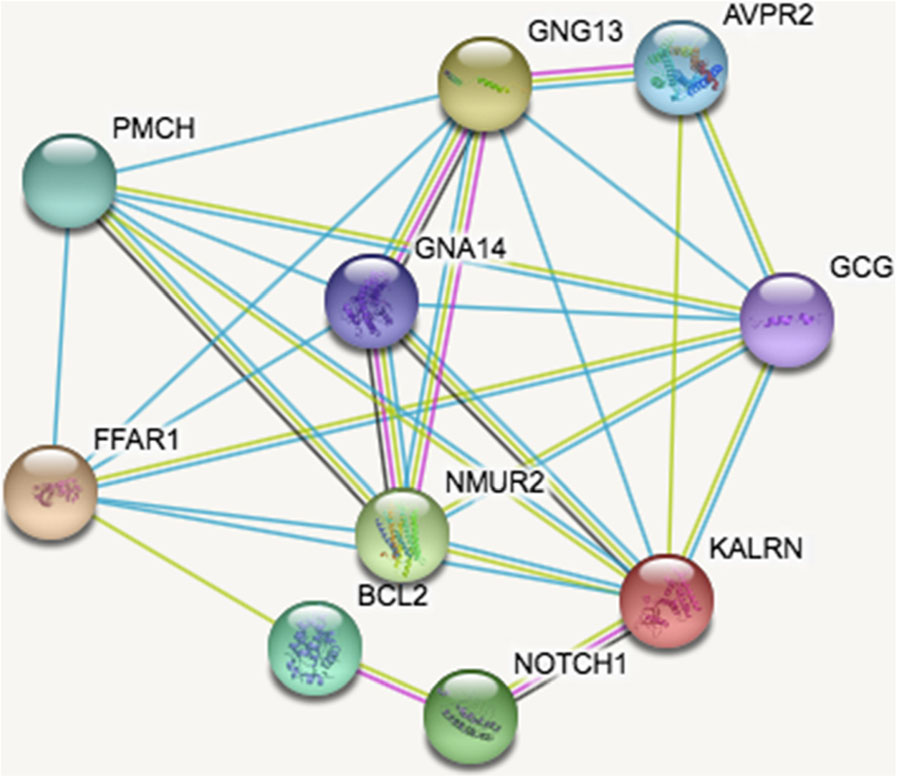
The interaction network of the top 10 core genes. The nodes indicated the top core genes, and the edges indicated the interactions between the core genes

The score of top 1 module including GCG, GNG13, NMUR2, and KALRN was 14, which had 14 nodes and 91 edges. The score of top 2 module including BCL2 and CD22 was 6, which had 6 nodes and 15 edges. The score of top 3 module including CBLB, RNF6, TRIM9, and FBXO44 was 6, which had 6 nodes and 15 edges. Lastly, the interaction network of the top 10 high-degree hub nodes (core genes) was made by STRING database in Fig. 5. GNG13, GCG, NOTCH1, BCL2, NMUR2, PMCH, FFAR1, AVPR2, GNA14, and KALRN, which regulate 7, 7, 2, 2, 6, 6, 6, 3, 6, and 7 targets, respectively, showed the good connectivity.

**Fig. 6:**
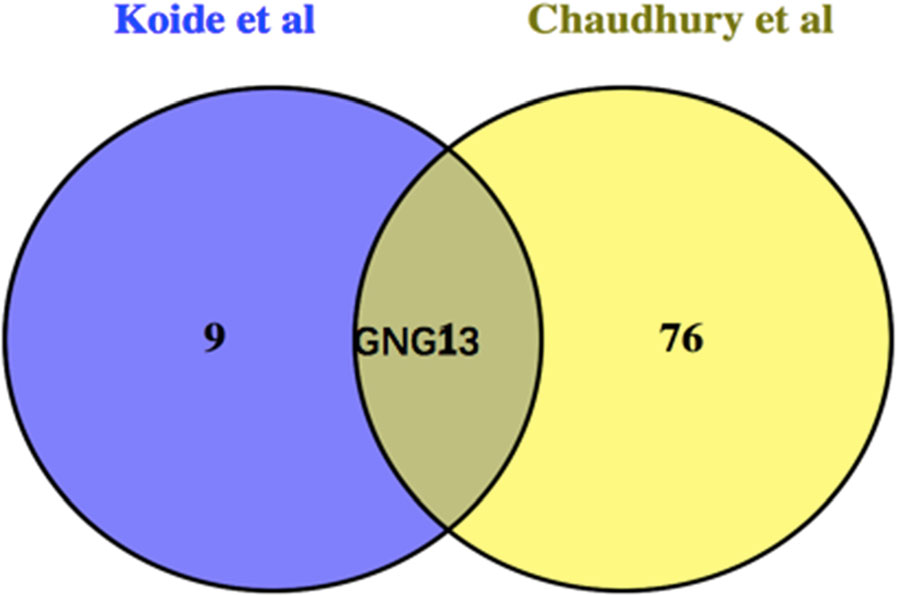
The Venn diagram

Interestingly, Chaudhury et al. [9, 11] reported that gene expression profiles of different-sized human rotator cuff tendon tears versus normal rotator cuff tendons. In order to seek the possibly common target genes, we pooled together the top 10 high-degree core genes as mentioned earlier and the 77 significantly DEGs of Chaudhury’s research using Venn diagram [9, 11]**.** GNG13 was discovered as the only common target gene in Fig. 6.

## Discussion

RCT is common and painful. Even after surgery, joint stability and function may not recover [11]**.** SCs play a major role in muscle regeneration. However, human SCs in muscles with atrophy, denervation, and fatty infiltration are unclear due to the difficulty in isolating from small samples, and the mechanism has not been elucidated [12–14]**.** In the present study, the gene expression profile of GSE93661 was downloaded and a bioinformatics analysis was performed. The results showed that there were 551 DEGs in SCs of torn rotator cuff tendons and normal rotator cuff tendons. Furthermore, GO, KEGG pathway, and gene–gene interaction network analysis were performed to obtain the biomarkers or the major genes related to pathogenicity mechanism of RCT.

In order to disclose the underlying molecular mechanisms between SCs and RCT, we characterized the possible GO functional terms and signaling pathways of DEGs. Considering the results of GO function analysis, we linked the DEGs with aging, regulation of ion transmembrane transports, ion channel activity, and calcium ion binding, which are very important for the development process of RCT. When muscle is injured, exercised, overused, or mechanically stretched, SCs are activated to enter the cell cycle, divide, differentiate, and fuse with the adjacent muscle fiber. In this way, SCs are responsible for regeneration and work-induced hypertrophy of muscle fibers. Ryuichi’s results suggested that the activation mechanism is a cascade of molecular events including an influx of calcium ions and their binding to calmodulin, nitric oxide synthase (NOS) activation, NO radical production by cNOS, matrix metalloproteinase activation, HGF release from the matrix, and presentation of HGF to the signaling receptor c-met. Understanding the mechanisms of SC activation is essential when planning procedures that could enhance muscle growth and repair [15]**.**

As previous articles reported, our KEGG pathway analysis showed that notch signaling pathway, hedgehog signaling pathway, dopaminergic synapse, GABAergic synapse, calcium signaling pathway, NF-kappa B signaling pathway, and estrogen signaling pathway were among the most relevant pathways for SCs in RCT. Pasut et al. found that in normal muscle, high levels of notch signaling is required to maintain the uncommitted state of SCs. Notch signaling plays a role in SC fate as activation of Notch1 strongly promotes the lineage switch from myogenic towards brown adipogenic fate [16]**.** Khayrullin et al. reported that upregulation of Notch signaling suppresses myogenesis and maintains muscle SC quiescence and miRNAs targeting Notch are likely to play important roles in alcohol-related myopathy in zebrafish model [17]**.** SC self-renewal is an essential process to maintaining the robustness of skeletal muscle regenerative capacity. Ogura’s study demonstrates that TNF-like weak inducer of apoptosis cytokine suppresses SC self-renewal through activating NF-kappa B and repressing Notch signaling [18]**.** Kamizaki’s findings indicate that Ror1 has a critical role in regulating SC proliferation via NF-kappa B activation during skeletal muscle regeneration of injured muscle [19]**.** In addition, estrogen regulates myosin heavy chain expression in SCs related to muscle function mainly through an estrogen receptor α-mediated pathway [20]**.** In Voronova's study, the formation of skeletal muscle during embryogenesis and adult muscle regeneration is regulated by myocyte enhancer factors and myogenic regulatory factors (such as MyoD). Hedgehog signaling could regulate MyoD expression during embryogenesis and adult muscle regeneration in SCs [21]**.**

The gene interaction network analysis revealed top 10 high-degree hub nodes of DEGs including GNG13, GCG, NOTCH1, BCL2, NMUR2, PMCH, FFAR1, AVPR2, GNA14, and KALRN. Most of them were not reported in SCs and RCT research. Only NOTCH1, who is a receptor that mediates intercellular signaling through a pathway conserved across the metazoan, had be studied [22]**.** Rando et al. found that activation of Notch1 signaling stimulates the proliferation of SCs and leads to the expansion of proliferating myoblasts. And, inhibition of Notch1 signaling abolishes SC activation and impairs muscle regeneration [23]**.** Also, recent studies found Notch1 is active in quiescent muscle SCs, and Notch1 signaling is critical for maintaining the quiescence of muscle SCs [24, 25]**.** As a supplement, Fujimaki et al. indicated that Notch1 and Notch2 coordinately maintain the SC pool in the quiescent state by preventing activation and regulate SC-fate decision in the activated state, governing adult muscle regeneration [26]**.** To sum up, upregulation of Notch1 may result in SC proliferation and self-renewal in the activated state, controlling muscle regeneration and improving muscle atrophy in RCT.

Furthermore, we analyzed the top 10 high-degree core genes and the 77 significantly DEGs of different-sized human rotator cuff tendon tears versus normal rotator cuff tendons in Chaudhury’s research using Venn diagram [27]**.** GNG13 was discovered as the only common core gene. Heterotrimeric G proteins, which consist of alpha, beta, and gamma subunits, function as signal transducers for the 7-transmembrane-helix G protein-coupled receptors. GNG13 is a gamma subunit that is expressed in taste, retinal, and neuronal tissues, and plays a key role in taste transduction [28]**.** Through KEGG pathway results, we discovered that GNG13 acts as an important node in dopaminergic synapse signaling, and targets PLC and AC5 in calcium signaling pathway. Many previous studies had emphasized the importance of calcium signaling pathway and skeletal muscle development, homeostasis, and regeneration. Calcium-ion is an important component of the signaling promoting muscle formation, muscle homeostasis, and regeneration. In particular, calcium-ion changes may direct muscle SCs to maintain their quiescent state, proliferate, or differentiate into functional muscle [29]**.** What is more, we are still learning how calcium signaling pathway and neuromuscular connections are restored on regenerating muscle. The establishment of connections between the motor nerve terminal and a post-synaptic region of membrane on regenerating fibers is essential to reinnervation and functional contractility, which is important for clarifying denervation atrophy pathological process of injured rotator cuff muscle. Recent researches have implicated SC signaling in the process of muscle reinnervation. SC have potential to influence axon growth and the reappearance of neuromuscular connections by their secretion of semaphorin 3A (Sema3A). Sema3A is a neural chemorepellent that is thought to coordinate the reconnection of motor axons with a differentiating fiber in a regenerating muscle. These direct proofs encourage a possible implication of SCs in the spatiotemporal regulation of extracellular Sema3A concentrations, which potentially ensures coordinating a delay in neurite sprouting and re-attachment of motoneuron terminals onto damaged muscle fibers early in muscle regeneration in synchrony with recovery of muscle-fiber integrity [30–32]**.** In addition, calcium signaling pathway is also linked with the activation of neuromuscular connections, and calcium-ion influx through voltage-gated calcium channels regulates the neuron’s responsiveness to Sema2A-dependent chemorepulsion exerted by the muscle [33]**.** Our research highlights the crucial role of nerve–muscle interaction in restoring innervation after RCT, and hypothesizes that SC-mediated GNG13 could affect neuromuscular connections and cause denervation atrophy via calcium signaling after rotator cuff muscle injured.

## Conclusions

In summary, 551 DEGs were identified including 272 upregulated DEGs and 279 downregulated DEGs screened in SCs of torn SSP samples compared with intact SSC samples. GO and KEGG pathway analysis provided a series of related key genes and pathways to contribute to the understanding of the molecular mechanisms between SCs and RCT, thus yielding clues to speculate the GNG13/calcium signaling pathway is highly correlated with the denervation atrophy pathological process of RCT. Furthermore, further experimental validation should be made in future studies.

## Abbreviations

BP: Biological process
CAMs: Cell adhesion molecules
CC: Cellular component
FC: Fold change
GEO: Gene Expression Omnibus database
GO: Gene ontology
HGF: Hepatocyte growth factor
KEGG: Kyoto Encyclopedia of Genes and Genomes
MCODE: Molecular Complex Detection
MF: Molecular function
NO: Nitric oxide
NOS: Nitric oxide synthase
RCT: Rotator cuff tear
SCs: Satellite cells
Sema3A: Semaphorin 3A
SSC: Subscapularis
SSP: Supraspinatus
STRING: Search Tool for the Retrieval of Interacting Genes
